# Environmental implications of lettuce sourcing: Comparison of sourcing from vertical farms and conventional production

**DOI:** 10.1016/j.heliyon.2024.e41503

**Published:** 2025-01-03

**Authors:** Aina Cabrero Siñol, Michael Martin

**Affiliations:** a Inèdit, C/ Diputació 37-39, Interior passage, Local 6B, 08015, Barcelona, Spain; b IVL Swedish Environmental Research Institute, Life Cycle Management, Sustainable Society, Vallhallavägen 81, 114 28, Stockholm, Sweden; c KTH Royal Institute of Technology, Department of Sustainable Development, Environmental Science and Engineering, Teknikringen 10B, 114 28, Stockholm, Sweden

**Keywords:** Vertical farming, Life cycle assessment, Supply chain, Logistics, Sustainable food systems

## Abstract

Today's globalised agricultural sector poses significant environmental challenges that are expected to worsen with population growth, increased urbanisation, and with the effects of climate change. In this context, vertical farming systems have gained traction as potential solutions to create a more resilient and sustainable food system. This study aims to evaluate the environmental performance of mixed salad bags from a conventional supply chain and compare it with that of mixed salad supplied by a large-scale vertical farm. Different locations for the vertical farm are also investigated. To compare the environmental impacts, life cycle assessment was conducted for various scenarios employing a cradle-to-grave perspective. The results suggest that the vertical-farmed supply can have lower environmental impacts compared to the conventional supply of mixed salad bags, e.g. having roughly 44 % lower CO_2_-eq emissions. However, in five of the eight impact categories assessed, the vertical farm was found to have higher emissions, notably for resource use. Furthermore, it was found that the location of the vertical farm can play a critical role in its sustainability due to varying electricity mix compositions and transportation distances to final processing or consumers. Furthermore, the results are sensitive to the conventional supply data for comparisons. The findings contribute to the growing field of vertical farming and provide valuable information for transitioning toward a more sustainable food system.

## Highlights

• Compares sourcing salad crops from conventional supply chains and vertical farms.
• Vertical-farmed products can have lower environmental impacts.
• Location of the vertical farm important due to electricity mix and transport.
• Results sensitive to conventional supply LCI data choices.
• Further datasets on conventional supplies are needed.

## Graphical abstract

**Figure:**
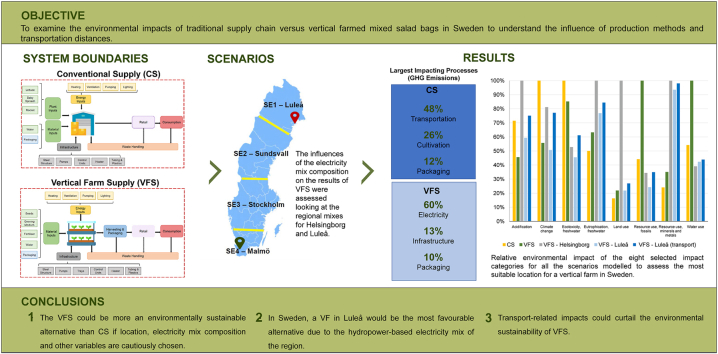

## 1 Introduction

Agriculture is one of the most environmentally taxing sectors in today's world as it contributes greatly to land degradation, biodiversity loss, and eutrophication. Moreover, research shows that more than a quarter (i.e., 26 %) of global greenhouse gas (GHG) emissions are caused by food production [1]. The sector is also responsible for 70 % of global freshwater withdrawals [2] and occupies 38 % of the global land surface (i.e., 1/3 cropland and 2/3 livestock pasture [3]). Other factors such as population growth, pandemics such as COVID-19, rapid urbanisation, climate change, and long supply chains add to the complexity of these sustainability challenges [4].

It is thus clear that more efficient supply chains and resource use are needed to maximise productivity and reduce the environmental impacts of our food system. Vertical farming (VF) has been gaining traction as a potential alternative agricultural practice, although its viability and profitability are currently limited [5]. Many different forms of VF exist but, in short, they can be defined as indoor crop production systems with multiple layers in which growth conditions are controlled [6]. Because production is independent of local weather conditions, these systems can guarantee constant quantity and quality of output everywhere in the world, and thus, they can shorten supply chains, which can lead to minimized food losses and increased shelf-life [6]. Furthermore, the multi-layer nature of VF is suggested to allow for the provision of a greater crop yield per square metre (m^2^) of land with a lower direct resource use compared with open-field (OF) production or greenhouses [7].

All these advantages, however, come at the expense of energy consumption. Technological advances such as improved light-emitting diode (LED) lighting have boosted the development of VF, yet great amounts of energy are needed to provide light and controlled conditions indoors. Indeed, the energy required is higher than in OF practices [6,8]. Therefore, this makes the environmental impacts of VFs highly dependent on the context-specific generation methods of the electricity mix where nearly all studies show the large influence of energy on the overall environmental performance of vertical farms [9,10]. Furthermore, as outlined in Martin and Orsini [10], there are few studies that assess the sustainability of vertical farms and compare their results to those of conventional supply chains. Assessing the effective sustainability advantage of shifting from the existing supply chains to VF is key for future developments. Empirical evidence from real case studies looking at more than just greenhouse gas (GHG) emissions is still missing, perhaps due to the evolving nature and novelty of the field [10,11]. As such, this paper aims to contribute to this knowledge gap and better understand the role VF could have in the existing food systems by comparing mixed salad supply from conventional supply chains to sourcing from vertical farms.

## 2 Materials and methods

The following sections describe the case study assessed, explain the methodology employed, and outline the additional scenarios and analyses conducted. Brief details regarding data collection are included, although further information is provided in the Supplementary Material.

### 2.1 Swedish lettuce provisioning

This research focuses on the provisioning of lettuce in Sweden as a case study. This country's high latitude makes a sufficient and stable year-round supply of local vegetables unattainable through conventional practices (e.g., open-field or polytunnel) since light intensity and temperature are not adequate for it [12]. According to Dahlberg and Linden [13], Sweden imports over 80 % of all fruits and greens it consumes, making the country heavily reliant on other economies. Moreover, Sweden has a largely renewable electricity mix, sourcing most of its electricity from nuclear (41 %) and hydro (39 %), along with a growing share of wind power (10 %) [14]. Therefore, since the literature shows that the environmental impacts of VF practices are highly dependent on the electricity mix composition, the country's energy profile makes it an attractive case study to compare VF to the conventional supply chain (notably based on imports).

### 2.2 Life cycle assessment

Life cycle assessment was used to evaluate the potential environmental impacts associated with the supply chain of mixed salad products over their full life cycle. Two product systems were compared. These include a scenario assessing a conventional supply chain (labelled CS) for packaged salad mix and an alternative supply chain sourcing a salad mix from a vertical farm (labelled VFS). The functional unit used is the fresh weight of mixed salad available to consumers, namely 1 kg (kg) of the edible portion of leafy greens cleaned and bagged for household consumption available at retail.

This study follows the procedure as outlined by ISO 14040:2006 and the Handbook on Life Cycle Assessment [15]. Moreover, since VFs are still in a premature deployment stage, the study employed an attributional approach with the aim to provide a snapshot of the environmental performance of the system without considering broader system-level effects or potential changes in consumption patterns or market dynamics [15], also following guidance from Ref. [10] for employing LCA to study the environmental performance of vertical farming.

The processes are modelled from a cradle-to-grave perspective, considering both upstream and downstream inputs and outputs, including fertiliser production, packaging, distribution, and waste handling processes. Transportation from the production site to retail is included, but transportation to household and energy related to storage are excluded. Cooking is not considered since the salad mix products (e.g., lettuce) are modelled as ready-to-eat (RTE), and the biochemical reaction in the human body (i.e., excretion) is defined as a cut-off. From the post-purchase stages, thus, only waste treatment (and transportation to the treatment site) are included in the system boundaries since it was considered important to assess the environmental impacts of the full life cycle.

Acknowledging the European Commission's efforts to enhance the comparability of LCA results, the ‘Environmental Footprint v. 3.0 Method’ (EF) impact assessment family is used. Although the method includes 28 impact categories, this study focuses only on eight of them as outlined in Table 1. These indicators, including acidification, carbon footprint, ecotoxicity, eutrophication, land and water use as well as resource depletion, are considered not only because they best reflect the environmental concerns for food systems, but also due to several authors highlighting their relevance in the assessment of vertical and urban farming alternatives [16,17].

**Table 1:** List of impact categories assessed, including their acronym, category indicator, and a brief description for each. Source [18]:

Impact category	Abbreviation	Category Indicator	Description
**Acidification**	AC	mol H+ eq	Refers to the potential acidification of soils and water caused by the release of gases such as nitrogen oxides and sulphur oxides
**Climate change**	CC	kg CO_2_ eq	Refers to the potential global warming due to emissions of GHG to the atmosphere.
**Ecotoxicity, freshwater**	ECF	CTU_e_	Refers to the impact of toxic substances emitted to the environment on freshwater organisms.
**Eutrophication, freshwater**	EUF	kg P eq	Refers to the nutrient-enrichment of freshwater ecosystems due to the release of nitrogen or phosphor-containing compounds.
**Land use**	LU	Pt	Refers to the changes in soil quality (Biotic production, Erosion resistance, Mechanical filtration). It is a dimensionless indicator, and thus the results are given in points (based on soil quality index).
**Resource use, fossils**	RUF	MJ	Refers to the depletion of natural fossil fuel resources.
**Resource use, minerals and metals**	RUM	kg Sb eq	Refers to the depletion of natural non-fossil resources.
**Water use**	WU	m^3^ depriv.	Refers to the relative amount of water used, based on regionalized water scarcity factors.

The product systems in this report are primarily based on data available in Ecoinvent cut-off database version 3.8 together with scientific literature and primary data gathered. Some processes are also based on LCI data from Agribalyse v. 3.1 and GaBi (i.e., Managed LCA Content database version 2023.1). The software used for the organisation and calculations is OpenLCA v1.11.0 (supported by Excel). Further details and motivations behind this choice are provided in the subsequent sections and in the Supplementary Material.

### 2.3 Life cycle inventory

#### 2.3.1 Conventional supply (CS)

The study evaluated the current supply chain of the Swedish company BAMA Fresh Cuts [19] as a case study to assess the life cycle environmental impacts of mixed salad bags. The company supplies the Swedish market with several RTE mixed salad bags. However, only four types of crops were considered in this study - namely iceberg lettuce, romaine, baby spinach, and arugula (accounting for roughly 57 % of BAMA's sold products). Although these leafy greens are different, they are assumed to be comparable since they have a similar nutritional value, and consumers often see them as substitutes as they are often sold in RTE mixed salad bags. As such, subsequent text refers to the different blends of leafy greens as RTE mixed salad bags. The company's modelled operational facility is located in Helsingborg and has a total area of 4200 m^2^, excluding canteen and offices, and yields an output of over 5800 tonnes of packaged RTE mixed salad bags annually.

**Fig. 1:**
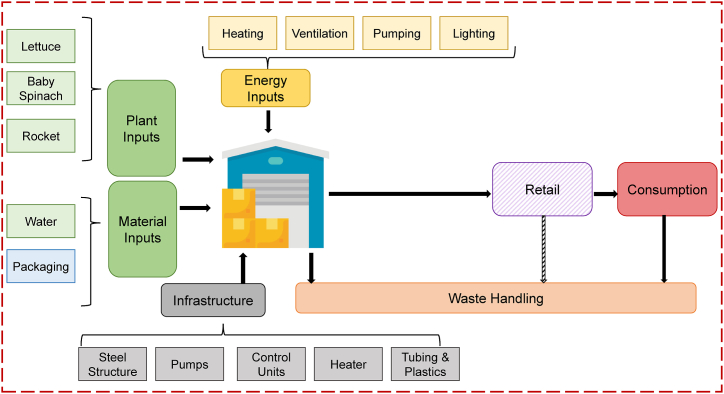
System boundaries for the Conventional Supply (CS) Scenario. Adapted from Martin et al. [11]. Retail and consumption impacts are excluded, but the end-of-life stage (i.e. waste handling) is included, hence a cradle-to-grave approach is taken.

Additional information regarding data collection and assumptions can be found in the Supplementary Material. Nonetheless, Fig. 1 below provides an overview of the system boundaries for the Conventional Supply (CS) scenario. Furthermore, Table 2 provides an inventory of all the inputs and outputs.

**Table 2:** Inventory of inputs and outputs for the Conventional Supply (CS) Scenario. Note: “Water” includes water to clean crops and the facility; and “Main Output” accounts already for the 3 % loss at retail.

Group (Aggregation)			Detail	Amount	Unit
**INPUTS**					
Material Inputs	Cultivation		Open field - lettuce	6 353 700	kg
			Open field - spinach	599 680	kg
			Tunnel	1 185 070	kg
	Cleaning	Chemicals	Potassium hydroxide	57	kg
			Sodium hypochlorite	400	kg
			Benzalkonium chloride	170	kg
		Water	Tap water	143 640 000	litre
	Packaging	Consum.	Polyethylene	24 370	kg
			Cardboard	48 730	kg
			Wood	79 800	kg
		Retail	Polyethylene	366 420	kg
Energy			Electricity	3 726 660	kWh
			Heating	634 350	kWh
Infrastructure		Structure	Aluminium	11 400	kg
			Steel	171 000	kg
			Tubing (PE)	180	m
			Assembly	182 400	kg
			Plastic	125 400	kg
		Other	Control Units	630	kg
			Pumps	9	units
			Other (electronics)	750	kg
			Cables	1140	kg
		Machinery	Robotics	830	kg
			Machines	22 170	kg
Maintenance			Travel	5700	km
			Steel	230	kg
			Control Units	23	kg
Transport - Facility (Helsingborg)			Salad imports - in	26 239 440	Tonne-km
			Consumables - in	52 000	Tonne-km
			Infra. & Maint. - in	2320	Tonne-km
			Waste Facility - out	136 170	Tonne-km
Transport – EoL			Product - retail	1 441 060	Tonne-km
			Shipment - retail	1 922 900	Tonne-km
			Waste EoL - out	26 850	Tonne-km
**OUTPUTS**					
Waste – BAMA			Biowaste	2 280 000	kg
			Wood	3990	kg
			Residual	294 690	kg
			Hazardous waste	1080	kg
			Wastewater	143 640	m3
Waste - EoL		Plastic	Packaging Recycling	183 210	kg
			Packaging not Recycling	183 210	kg
		Biowaste	Composted	85 240	kg
			Not composted	85 240	kg
Co-products			Cardboard	47310	kg
			Plastic	1200	kg
Main Output		Mixed Salad Bags	Available at retail∗	**5 682 700**	kg

#### 2.3.2 Vertical farm supply (VFS)

**Fig. 2:**
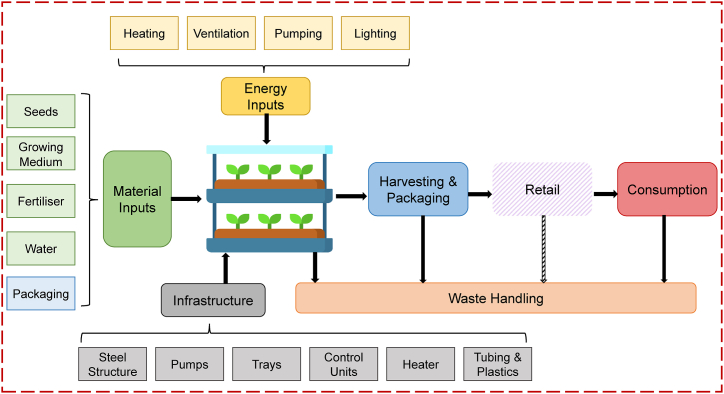
System boundaries for the VFS scenario. Adapted from Martin et al. [11]. Retail and consumption impacts are excluded, but the end-of-life stage (i.e., waste handling) is included, hence a cradle-to-grave approach is taken.

The vertical farm scenario was modelled based on data from Martin et al. [11]. This study provided a good reference for data since the article looks at the largest commercial and operational hydroponic vertical farm in Sweden, i.e., Ljusgårda. Although the facility of the actual VF is based in Tibro, the modelled site was assumed to be in Helsingborg since it is where BAMA's operations are based. This choice allowed for more accurate comparison between the CS and the VFS, making transportation distances to retail the same for both cases. The modelled VF has a total area of 7000 m2 of floor space, with approximately 25 % dedicated to the production, and yields an output of 520 000 kg of mixed salad bags annually, which are packaged at the vertical farm. Fig. 2 below provides an overview of the system boundaries for the VFS, and Table 3 shows its inventory of inputs and outputs. Additional information regarding data collection and assumptions can be found in the Supplementary Material.

**Table 3:** Inventory of inputs and outputs for VFS. Note that “Main Output” already includes the 3 % loss at retail. In addition, Water also includes water used to clean the lettuce and the facility.

Group (Aggregation)			Detail	Amount	Unit
***INPUTS***					
Material Inputs	Growing Media		Peat (Black)	60 670	kg
			Coconut coir	60 670	kg
			Clay	86	kg
	Seeds		Lettuce Seeds	86	kg
	Fertilisers		KNO_3_	2240	kg
			MgSO_4_	410	kg
			KH_2_PO_4_	810	kg
			K_2_SO_4_	9450	kg
			CaNO_3_	2200	kg
			MgNO_3_	7450	kg
			CaCl_2_	62	kg
			Iron (Fe)	9.52	kg
			Calcium (K)	6.66	kg
			Manganese (Mn)	4.76	kg
			Boron (B)	1.90	kg
			Zinc (Zn)	1.19	kg
			Mn (Molybdenum)	0.36	kg
			Copper (Cu)	0.24	kg
	Carbon Enrichment		CO_2_	54 000	kg
	Irrigation		Tap Water	4 723 000	litre
	PH Adjust		Phosphoric Acid	1 3340	kg
	Cleaning		Tap water	792 000	kg
	Packaging	Consum.	Polyethylene	30	kg
			Cardboard	300	kg
		Retail	Polyethylene	32 520	kg
Electricity			Total used	5 160 000	kWh
Infrastructure	LEDs		Aluminium	1220	kg
			Diodes	80	kg
			HDPE (plastic)	150	kg
			Wire	80	kg
	Growing Infra.	Structure	Aluminium	4140	kg
			Steel	59 710	kg
			Plastic	43 740	kg
			Assembly	63 850	kg
			Polypropylene	1690	kg
		Other	Control Units	200	kg
			Pumps	10	units
			Other/Sensors	270	kg
			Cables	400	kg
		Machinery	Machines	28 100	kg
			Robotics	290	kg
Maintenance			Travel	500	km
			Steel	200	kg
			Control Units	20	kg
Transport - VF			Consumables - in	21 710	Tonne-km
			Infrastructure - in	2670	Tonne-km
			Waste Farm - out	8140	Tonne-km
Transport – EoL			Product - retail	127 910	Tonne-km
			Shipment - retail	170680	Tonne-km
			Waste EoL - out	2380	Tonne-km
***OUTPUTS***					
Waste - Farm	From consum.		Biowaste	136 930	kg
			Packaging - Plastic	360	kg
			Cardboard	300	kg
			Wastewater	890	m3
	From infra.		Infra. - Plastic	22 070	kg
			Electronics	80	kg
			Metal	3070	kg
Waste - EoL	Plastic		Packaging recycling	16 260	kg
			Packaging not recycled	16 260	kg
	Biowaste		Composted	7570	kg
			Not composted	7570	kg
Main Output	Mixed salad bags		Available at retail∗	**504 400**	kg

### 2.4 Scenario development

**Fig. 3:**
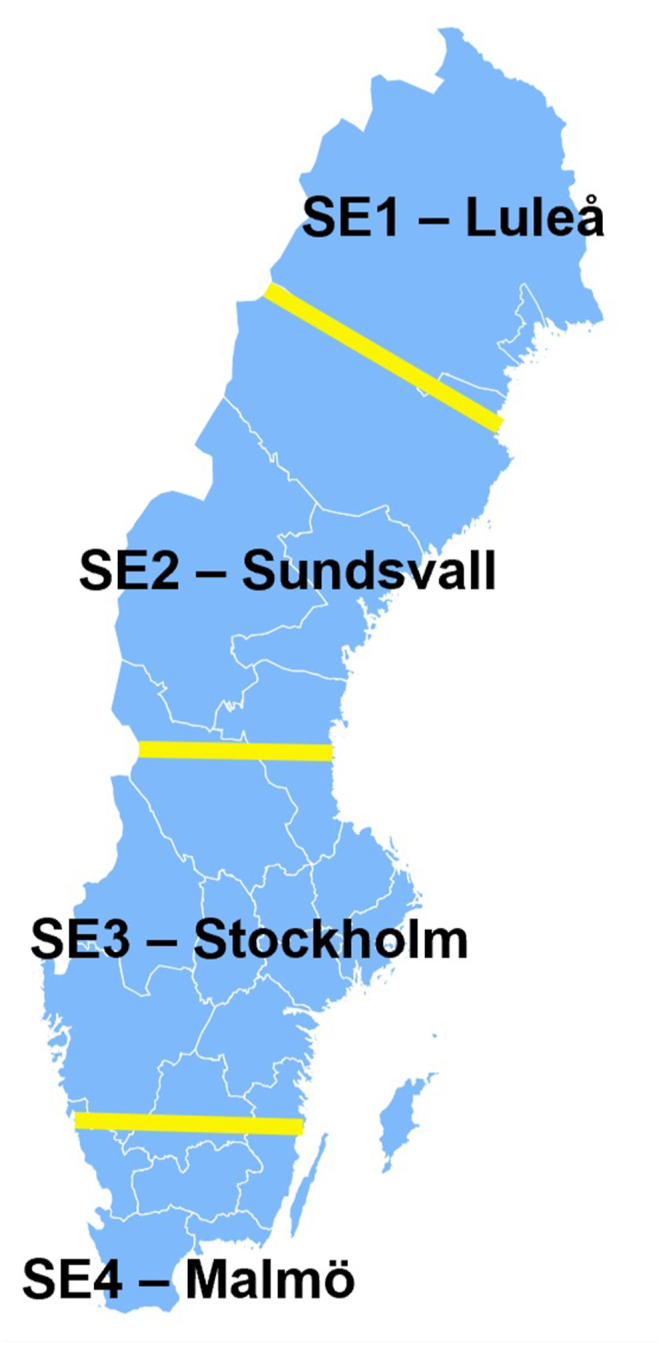
Electricity mix composition of the four bidding areas of the Swedish electricity system in 2018. Data from Refs. [22,23].

As mentioned, the general or average national electricity mix of Sweden was used to model the baseline scenario for both the CS and VFS scenarios. However, the Swedish electricity mix presents significant differences domestically. These differences are noticeable when looking at the four bidding areas from Svenska Kraftnät, which are divided into – SE1 - Luleå, SE2 - Sundsvall, SE3 - Stockholm, and SE4 – Malmö (Fig. 3). Additionally, as mentioned, literature shows that the environmental impacts of vertical farming systems are highly dependent on electricity mix composition; see e.g. Refs. [11,20,21]. Therefore, to study the implications of locating the vertical farm in different areas in Sweden, the impact of the country's regional variations is studied by modelling two scenarios for the VFS with the regional mixes presenting the biggest differences in composition, i.e. SE4 and SE1.

To more accurately assess which location would have lower environmental impacts, transportation distance changes were also included when considering locating the vertical farm in Luleå. As such, travel distance was increased by 350 km for the retail stores in the central part of Sweden and by 1340 km for the retail stores in the south.

## 3 Results and analysis

This section aims to evaluate, understand, and compare the potential environmental impacts of the studied product systems. This is done by comparing the characterisation results of the baseline scenario (CS), presenting their contribution analyses, and assessing the differences between the proposed scenarios for the VFS. Thereafter, sensitivity analyses are conducted to assess the robustness of the findings.

### 3.1 Baseline comparison between the VFS and CS

**Fig. 4:**
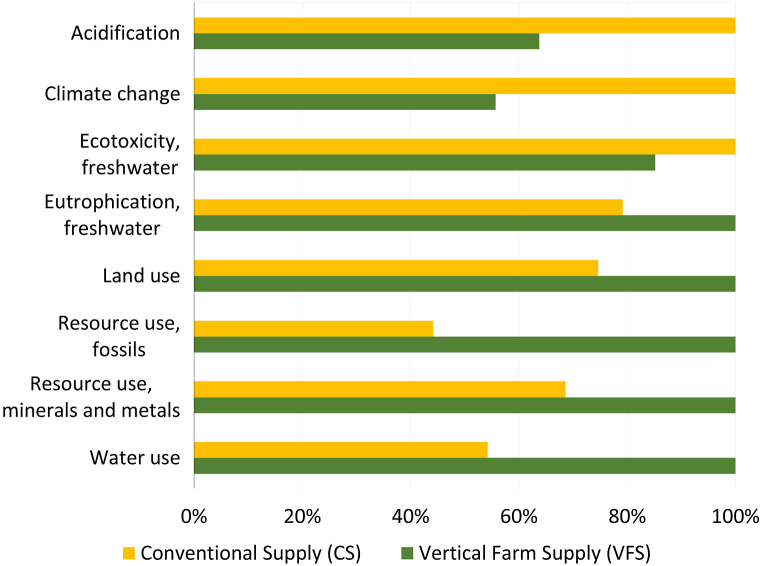
Relative environmental impact of CS and VFS. For numerical details refer to Supplementary Material.

Fig. 4 shows the environmental impacts of the CS and VFS scenarios relative to the highest value of the impact category to better compare both supply chains. Further details can be found in the full characterisation tables for the VFS and CS in the Supplementary Material (Tables S4 and S5).

It can be observed that the VFS has larger environmental impacts in five out of the eight impact categories assessed while the CS does only in three. The VFS was found to have lower GHG emissions, potential acidification, and ecotoxicity to freshwater. Although in vertical farming the direct use of water and land is often found to be lower than in traditional cultivation methods such as open field [6,9,24,25], the life cycle-based water use and land use impacts were found to be higher for the VFS scenario. This fact, along with larger emissions in other categories assessed, can be explained by the indirect impacts of the high electricity consumption in the VFS using the Swedish grid electricity containing a large share of hydropower, see Fig. 6 below. Thereafter, the categories of water use, land use, freshwater eutrophication, resource use fossil, and resource use mineral and metal categories are higher in the VFS scenario.

#### 3.1.1 Conventional supply results

**Fig. 5:**
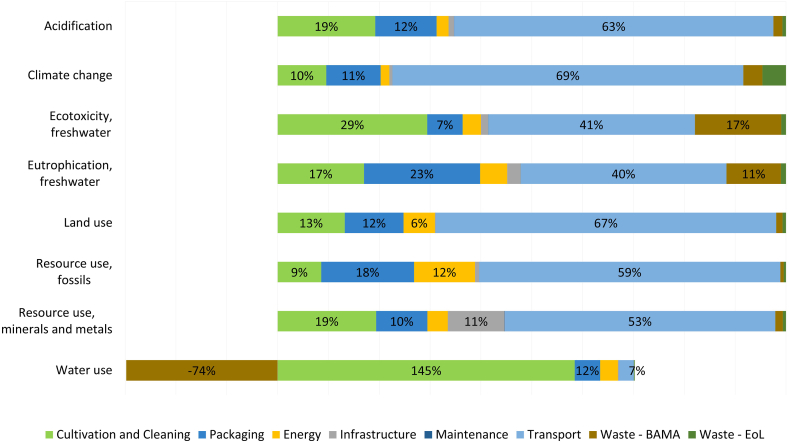
Cradle-to-grave contribution of life-cycle stages to the environmental impacts of 1 kg of mixed salad bags for the Conventional Supply (CS). Data labels are only provided for processes contributing to more than 5 % in each impact category.

For the CS scenario, Fig. 5 shows that transportation contributes between 40 % and 69 % to all the impact categories except for WU. The combustion of fossil fuels from the modelled road transport causes harmful exhaust gases to leak into the environment, which contribute to several impact categories – CO_2_ to CC, aluminium and hydrocarbons to ECF, nitrogen oxides and sulphur dioxide to AC and phosphate to EUF. Moreover, not only extensive areas and (fossil and mineral) resources are needed to produce fuels and vehicles but also for the construction of road networks (i.e., affecting RUF, RUM and LU). As such the transportation-related impacts result in the CS scenario being more environmentally taxing than VFS in the categories AC, CC, and ECF.

**Fig. 6:**
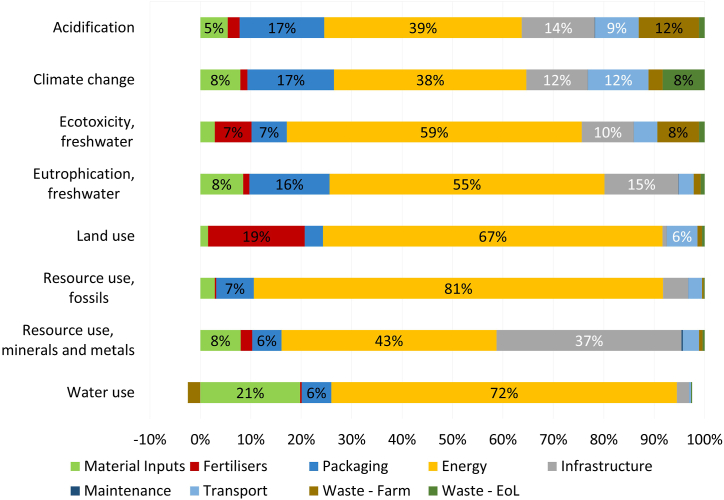
Cradle-to-grave contribution of life-cycle stages to the environmental impacts of 1 kg of lettuce bagged and cleaned for the VFS System II – VFS. Data labels are provided for processes contributing to more than 5 % in each respective impact category.

Cultivation and cleaning is another dominant group across all impact categories. It has the highest impact in WU due to irrigation and the water used for cleaning. Thereafter, it has the second highest in AC and ECF stemming from ammonia and nitrogen oxide flows during cultivation, among others. Cultivation-related impacts in the CS were, therefore, found to be more environmentally taxing than VF in AC and ECF. Packaging is another relevant category, ranking second in EUF and RUF with 23 % and 18 % of the impacts, respectively. Moreover, it is worth noting that Energy (primarily electricity) used at the facility in Helsingborg accounts for 12 % of RUF, which is roughly 0.75 kWh per 1 kg of packed mixed salad.

In Fig. 5, a negative contribution of 73 % from Waste is illustrated. This is because the water employed is sent to a treatment facility, and most of it is restored to the same water basin from which it was initially extracted, reducing the depletion effects of its use. It is important to bear in mind, however, that this cannot be interpreted as a beneficial effect of the product system; similar assertions can also be drawn for the VFS. The annual water used at the facility is still very large, roughly 150 million litres, and the treatment of this water has other environmentally negative effects, such as increasing ECF. Other waste treatment-related activities at the packaging site, i.e., BAMA, also have a significant contribution to ECF and EUF (i.e., 18 % and 11 %, respectively) attributed to processes such as industrial composting or plastic recycling.

#### 3.1.2 Vertical farm system results

Electricity required at the VF, primarily for lighting and cooling, was found to be the largest contributor for all categories (i.e., 10 kWh required per kg of lettuce). Its shares range from 81 % to 38 %, the highest category being RUF and the lowest CC. For GHG emissions, electricity-related impacts contributed to over half of the burdens, which were attributed mainly to the share of thermal power in the electricity mix of Sweden (i.e., 9 %), namely emissions caused by the combustion of hard coal, natural gas, and crude oil. Biomass is also widely used as a fuel in the country's thermal power plants, which was found to be the driver behind the contribution of electricity to the land use impacts in the LU impact category (i.e., 68 %, mainly due to the acquisition of wood chips causing the occupation and transformation of forest areas). On the other hand, the uranium used in nuclear plants accounts for almost all the RUF-energy-related impacts, again as the Swedish electricity mix contains roughly 40 % nuclear energy. Although it is classified as a heavy metal, this radioactive resource has a very high characterization factor for the RUF category (i.e., 560 000 MJ/kg using EF 3.0 Method), resulting in the VFS being more environmentally taxing than CS for the RUF category.

Packaging, Infrastructure and Transport also account for relatively large shares in several categories. Packaging, and especially low-density polyethylene used as bagging film at retail, was found to drive AC, CC and EUF, contributing to an average of 17 % of the total impact in these categories. Transport primarily contributed to AC, CC, and LU. Resources employed for building the VF's structure (e.g. aluminium, copper or chromium) influenced RUM by 38 %. Fertilisers were found to contribute to over 7 % of the impacts in ECF, with an additionally large effect (i.e., almost 20 %) in LU stemming mainly from the mining of potassium nitrate. Material inputs (which include water for irrigation) contributed roughly 20 % to WU and roughly 72 % for energy. The negative contribution to WU, shown in Fig. 6, was again owed to wastewater treated externally as explained for the CS baseline. Waste (VF and EoL together) accounted for an average of over 11 % in AC, ECF, and CC, mainly due to the treatment of biowaste, emitting ammonia and hydrogen sulphide to the atmosphere, and the recycling of plastic releasing GHG emissions.

### 3.2 VFS location scenario analysis

To assess the implications of the location of the vertical farm, and the associated energy mixes in the regions, an analysis was conducted to study the environmental performance in northern Sweden, with an additional scenario to include the transportation of the products to the processing location in the south of Sweden. As Table 4 suggests, the different electricity mixes largely affect the overall environmental impact of the VFS. On the one hand, the results show that the electrical mix (Zone 4 as shown in Fig. 3) of Helsingborg foremost increases the impacts on LU, RUM, and AC by 357 %, 185 %, and 119 %, respectively, potentially owing to a higher share of thermal (i.e., 11 %) and wind power (i.e., 50 %). These energy sources require the production of wood chips and the mining of minerals such as steel, aluminium and REEs. The relevant decrease in RUF and WU could be motivated by the decreased share in nuclear power requiring less uranium and less water for cooling purposes (i.e., no nuclear power is used in Helsingborg's region). RUM, RUF, and WU showed similarities when employing Luleå’s electrical mix, increasing 167 % and decreasing 58 % and 76 %, respectively, in comparison with the baseline scenario due to the increase of renewables (in this case hydropower, with a share of 91 % in Luleå) and the null share, again, of nuclear.

**Table 4:**
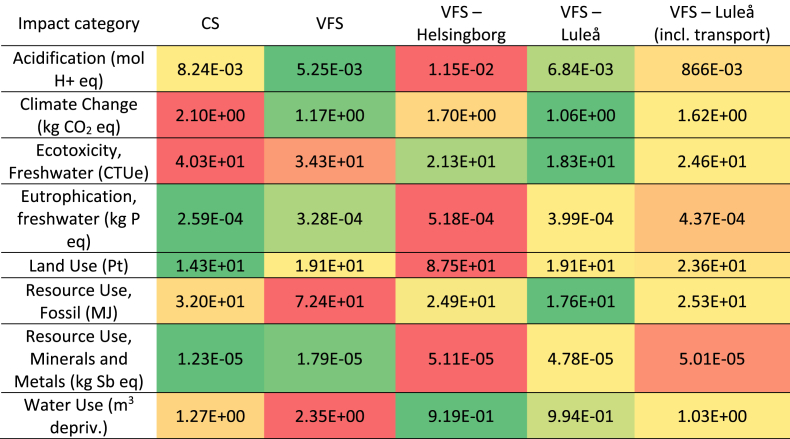
Sensitivity for the VFS results employing different regional electricity mixes in addition to additional transport distances to show the implications of the location.

Overall, the results portray the VFS in Luleå as the most environmentally sound option with a lower impact than CS on five out of the eight impact categories assessed. When accounting for the extra food mileage however, the results revealed an increase in environmental burdens for all categories making VF only better than CS in half of the categories. The most relevant increase for on CC and RUF (i.e. 52 % and 44 %, respectively) owing to the combustion of fossil fuels as it is assumed that lorries are fuelled by 100 % diesel, although Sweden has large shares of renewable fuel in the transportation mix.

### 3.3 Sensitivity analysis to LCI data

As the comparison to conventional supplies relies on LCI data from databases, the dataset employed can be an area of sensitivity for the results and comparisons. As such, for the sensitivity analysis, the Agribalyse dataset ‘lettuce, open-field, conventional, at farm gate was instead employed to represent the lettuce share for the CS scenario. Similarly, for the polytunnel-supplied lettuce, the Ecoinvent process ‘Lettuce360 production, in heated greenhouse’ was used to study the implications of using this dataset instead of that used in the baseline. Further details of the datasets employed are provided in the Supplementary Material.

**Fig. 7:**
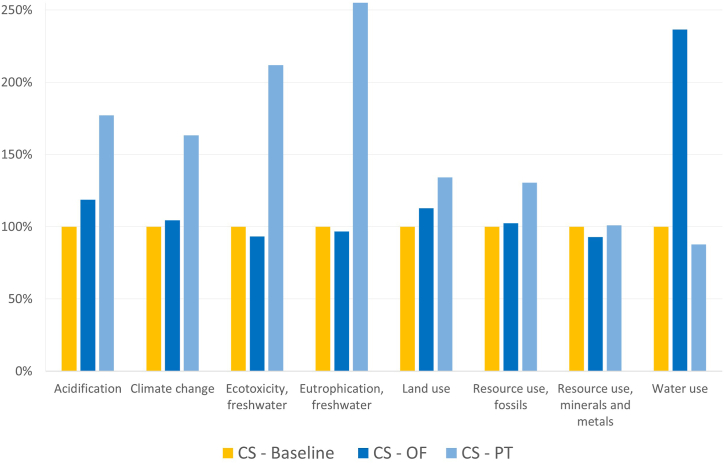
Sensitivity to the LCI choices to model lettuce. CS-OF (Conventional Supply employing LCI data from Agribalyse). CS-PT (Conventional supply employing Lettuce360 dataset from Ecoinvent to model polytunnel production). All impacts for the CS-Baseline scenario are set to 100 % to show the implications of different LCI dataset choices.

As shown in Fig. 7, the results for the CS are largely sensitive to the LCI dataset chosen to represent the conventional supply. From the CS-Baseline, the use of the dataset for open field production (CS-OF) results in only minor increases and decreases for all the environmental impacts assessed, with exception to a much larger water use impact. However, if instead the dataset for lettuce produced in a polytunnel was replaced with the Lettuce360 dataset from Ecoinvent, the environmental impacts show large increases in nearly all impact categories, except for resource use and lower water use, owing to the greenhouse technology.

## 4 Discussion

### 4.1 Comparing VFS and CS

This study suggests that the production of mixed salad (primarily lettuce) can be less environmentally impacting if produced in a vertical farm under the right circumstances. The results, however, have proved to be sensitive to modelling choices. For example, the location of the vertical farm can be of importance to reduce the environmental impacts based on the regional electricity mix as shown when comparing locating the vertical farm in the north or south of Sweden respectively. Similar results for regional differences can be found in several previous studies where the environmental performance of vertical farms and food production processes varies largely on location [[26], [27], [28]].

The electricity required at the VF was found to be the largest contributor to all impact categories. This supports similar results found in previous research [6,11,29]. However, the lesser contribution of electricity consumption in this study to the overall system's impacts versus the results found in the literature must be acknowledged. Research claims that electricity requirements contribute more than 90 % to the overall environmental burdens of CEA systems [21,29]. However, this study finds that its average contribution is at 57 % (only 38 % to CC). The reasons behind this could stem from Sweden's electricity mix composition, largely based on renewables, or from the scope of most comparative studies, which often take a cradle-to-gate perspective excluding the impacts related to transportation (to retail) and the product's end of life. Several studies also claim that transportation accounts only for a small share of the carbon footprint of a product, namely 4.8 % of the total food-system GHG emissions [30]. Although this concurs with the contribution of transport to the environmental impacts of the VFS (i.e., average of 5 %), it does not conform with the results of the CS scenario (i.e., average of 50 %). Yet, for this case more product-specific research was found showing that impacts increase when looking at easily perishable products such as salad crops [1]. Also, Sandison et al. [21] find transportation to account for 42.5 % of the overall CC impact category when studying the supply chain of lettuce employing open-field practices. Consequently, for CS, Cultivation&Cleaning, and Packaging were found to be the next two most contributing processes, with average shares of 33 % and 13 %, respectively, across all categories in line with existing literature. Milá i Canals et al. [31] claim cultivation to be a dominant stage in the supply chain of salad crops while previous research finds packaging to be another relevant category in traditional supply chains [1,11,30]. Similarly, the significant shares of Infrastructure, Packaging and Transport, comply with the findings of [11].

The Cultivation impacts of CS were found to be pointedly different when compared to other results existing in the literature. An example being the numbers for field production in Spain per kg of lettuce found in Refs. [9,31], where CC and WU impacts amounted 0.68 kg of CO_2_ eq and 13 m^3^ of water deprived, respectively, which largely differ from the results found in this study (i.e. 0.29 kg of CO_2_ eq and 2.52 m^3^ of water deprived per kg of lettuce). Specific regional environmental conditions, technology used, or resources employed could be some of the reasons influencing these results, e.g., in the LCI datasets employed. An example could be the difference in water scarcity between regions of cultivation; in 2020, Spain and Italy (which was the source of more than 50 % of the salad for the CS) scored 43 % and 30 % on the UN SDG Indicator 6.4.2 (Water Stress) thus showing high water scarcity [3]. However, since most of the salad was modelled using Agribalyse cultivation datasets, the location was assumed to be France, where the water stress indicator is lower (i.e., 23 %). This shows that more precise data regarding Spanish and Italian production of salad would potentially increase the WU for production (similar to Ref. [9]). Other factors that could affect WU impacts include circularity measures for the reuse of water at the facilities, collection of rainwater or the implementation of technologies capturing condensed water. They were not addressed in this study but may be interesting to assess in further research.

Another factor to be considered when comparing the environmental performance of CS with VFS is the generation of waste. The controlled conditions at the VF, the elimination of a cleaning step, and the reduced transport distances of the proposed alternative are factors that can contribute to a decrease in the amount of biowaste generated by today's long supply chains. In this study, when comparing the biowaste generated at both facilities (for CS and VF), a reduction of 32 % per kg of lettuce was found. Therefore, accounting for this added benefit for VFS would enhance its environmental performance against CS.

### 4.2 Limitations

Modelling choices, especially for the processes contributing the most to the system's overall impacts, could potentially influence results, as seen with the sensitivity analyses conducted. The Conventional supply scenario (CS) scenario was found to be sensitive to the choice of cultivation datasets. Similarly, transportation was modelled using processes from Ecoinvent 3.8 since no primary data was available. These processes assume lorries to be fuelled by 100 % diesel and thus neglect the potential benefits brought by the use of biofuels. According to a study assessing herb production in greenhouses [20], the use of biodiesel combined with hydrotreated vegetable oil could reduce environmental impacts such as acidification and CC. Transportation impacts only contribute an average of 5 % in VFS and thus, the impact of using biofuels is unlikely to be a determinant factor. However, in the CS, they account for 50 % on average, hence, it would be interesting to assess the environmental performance improvement of the CS if these alternative fuels were used. In Sweden, transportation is already using a larger share of biofuels (i.e., a minimum of about 10 %), but more detailed data regarding other regions or companies providing this service was unattainable. Therefore, the collection of primary data from the case-specific suppliers of crops and the fuel types used in logistics would be beneficial in increasing the internal validity of the present study.

Other limitations of this study are the proxies used to represent the different crops processed at BAMA's facility for the mixed salad bags (i.e., iceberg lettuce, romaine, arugula, and baby spinach) to the Ljusgårda's output, which are two varieties of crisp lettuce. These assumptions dwindle the accuracy of the study since VFS currently only produces a limited range of profitable crops (e.g., some leafy vegetables, herbs, and microgreens [11,32].

Moreover, regarding external validity, it is important to mention that this study focuses on a specific case study in Sweden based on data from Ljusgårda and BAMA data and is therefore not necessarily representative. Strong conclusions should not be generalised, and careful consideration to details should be taken when making comparisons with other studies. For instance, the modelled VFS requires 10 kWh per kilogram of lettuce produced while the average VFS in literature uses roughly 20 kWh/kg [33]. Furthermore, it is important to note that only an environmental perspective was taken in this study, thus neglecting the other two aspects from the triple bottom line of sustainability (i.e. economic and social). For CS, for instance, a significant amount of human labour is required to run operations such as sorting at the facility in Helsingborg. Changing the CS to a VFS could have an impact on the employment and working conditions of the modelled systems. Other aspects such as the initial investment and the operational costs of running a VF should be further assessed to understand the economic potential of realising such a supply chain shift. Moreover, consumer acceptance could also play a crucial role in the successful deployment of vertically farmed products, and it is not addressed in this report. Although VFS may help contribute to a more stable and less environmentally straining provision of certain foods, the controlled conditions during cultivation may be perceived as artificial or disconnected from nature, creating resistance and skepticism among consumers [34]. Research shows that, although VF systems are not broadly accepted by Nordic consumers, their potential contribution towards increased sustainability of food systems is among the main forces driving consumer acceptance [35]. There are also large possibilities to improve vertical farms by altering the environmental conditions (e.g., lighting, energy, fertilizers, etc.), and as such, the study employed could have improved impacts compared to those employed. Thus, more holistic research is needed to promote the sustainability benefits of the deployment of such alternative practices.

## 5 Conclusion

By performing a life cycle assessment on the conventional supply chain of mixed salad bags in Sweden and a vertical farm supply alternative, this research aimed to evaluate the environmental impacts associated with both systems and discover under which circumstances one option would be more environmentally sound. The findings revealed that transportation, the cultivation of crops, and packaging materials contributed the most to the overall environmental performance of CS with an average contribution of 50 %, 33 % and 13 %, respectively, to all impact categories. Electricity needed for lightning and ventilation of the VFS accounted for roughly 57 % of all impact categories addressed, thus being the single highest contributor. Subsequently, the infrastructure for the vertical farm, packaging, and other material inputs were found to be significant processes for the VFS with shares of 12 %, 10 %, and 7 %, respectively.

When comparing the systems' baseline scenarios using the general electricity mix of Sweden, it was found that CS performed best in five of the impact categories under study (i.e., EUF, LU, RUF, RUM and WU) and VFS only in three (i.e. AC, CC and ECF). These differences were primarily driven by the highly contributing processes mentioned. CC, for instance, was found to be 2.10 kg of CO_2_ eq per kg edible salad mix for the CS and 1.17 kg of CO_2_ eq per kg for VF (i.e., per kg of mixed salad) owing to the higher GHG emissions in the CS from transport (diesel combustion) and (plastic) packaging. The results for the RUF instead yielded values of 32 MJ for CS and 72.4 MJ for VFS (per kg of salad mix) due to the use of uranium for nuclear power, which accounts for 41 % of the Swedish electricity grid. When different regional electricity mixes were considered, it became clear that the environmental sustainability of VFS is highly dependent on grid composition. When using the electricity mix of Luleå, VFS performed better than CS, whereas applying Helsingborg's regional mix resulted in a tie. Nonetheless, the electricity mix proved not to be the sole main factor skewing the results since including the increased transportation distances required to deliver the mixed salad products to retail increased the impacts of the VFS when it was in the north of Sweden.

In short, despite a few limitations, the present study shows how the VFS could be more environmentally sound compared to conventional sourcing for several impact categories including acidification, climate change, ecotoxicity and eutrophication of freshwater, land use and fossils' resource use. Nonetheless, careful consideration of location and other initial choices (e.g., materials used for infrastructure and packaging or fuels used in transportation) is key for the improved environmental performance of this innovative cultivation practice and thus must be done cautiously before its implementation and deployment. Other aspects of the studied supply chains, such as the social and economic perspective, should be explored in future research to make more holistic and robust inferences about the systems’ sustainability. Moreover, since the results of this study are sensitive to dataset choices, the acquisition of more specific data such as regionally specific environmental impacts of different crops and cultivation practices (e.g. polytunnel cultivation of crops in Italy) and fuel types used in transportation logistics should be developed. Altogether, this study can be seen as an initial step in the sustainability assessment of a supply chain for mixed salad bags supplied by vertical farming through the evaluation of a real case study. Future work can build on the analyses conducted in this research, thereby clarifying under which circumstances vertical farming is environmentally and economically favourable compared to the conventional supply chain for mixed salad.

The findings of the study could stimulate interest and investment in vertical farming as a viable and environmentally friendly method for lettuce and salad crop production. Similarly, policymakers and industry stakeholders such as farmers and consumers may use these LCA findings to inform decision-making processes and support the development of policies and incentives for vertical farming, also allowing for more informed sourcing for lettuce and other salad crops. The present study could influence agricultural and environmental regulations, as well as promote the integration of vertical farming into sustainable food production and consumption strategies.

## CRediT authorship contribution statement

**Aina Cabrero Siñol:** Writing – review & editing, Writing – original draft, Visualization, Methodology, Investigation, Formal analysis, Data curation, Conceptualization. **Michael Martin:** Writing – review & editing, Writing – original draft, Visualization, Validation, Supervision, Software, Resources, Project administration, Methodology, Investigation, Funding acquisition, Formal analysis, Data curation, Conceptualization.

## Funding

Support for this work was provided by the 10.13039/501100004359Swedish Research Council for Sustainable Development -FORMAS through grant 2022-02036, and the Swedish Innovation Agency-Vinnova through grant 2019-03178.

## Declaration of competing interest

The authors declare that they have no known competing financial interests or personal relationships that could have appeared to influence the work reported in this paper.
