# Supplementary material for: Environmental implications of lettuce sourcing: Comparison of sourcing from vertical farms and conventional production
Source: Heliyon. 2025 Jan 3;11(1):e41503. doi: 10.1016/j.heliyon.2024.e41503 (PMC11759643; doi:10.1016/j.heliyon.2024.e41503)
Supplement: Multimedia component 1 [file mmc1.docx]

SUPPLEMENTARY MATERIAL

# Baseline Scenarios: Methodological Choices

For the quantification of the system’s environmental impacts, the collection of data is necessary. In this study, primary data was gathered through expert consultation (i.e. Anna Fråne and Michael Martin) and complemented with a site visit to the BAMA Fresh Cuts’ facility in Helsingborg. Secondary data was taken from scientific literature and existing databases (i.e. mainly Ecoinvent v. 3.8, but also Agribalyse v. 3.1 and GaBi). When no data was available proxies or cut-offs were used. Proxies, however, are not very precise as they incorporate data that has not been collected for the process they are representing. Therefore, to assess the influence of such modelling choices, sensitivity analyses have also been conducted. For instance, perturbation was assessed for the datasets used as cultivation processes in System I (i.e., where proxies were used). The paragraphs and subsections below aim to report and argue more in detail about the modeling choices made for this study. Some of the data provided by BAMA Fresh Cuts (e.g. revenue), however, has not been disclosed as requested by the collaborating party.

Regarding all background processes, data based on the geography of Sweden was prioritised when available and Europe or Global scope was used otherwise. For the inputs, market activities (i.e. labelled as “market for” in Ecoinvent 3.8) were used to represent the consumption mix of a product,
 linking product-specific suppliers with consumers within a specific geographical area, accounting for transportation and, if relevant, imports and losses as well (Ecoinvent 3.8, 2021). The waste outputs were linked to Ecoinvent processes given as treatment activities. Excluded from both systems were retail facilities, energy related to storage and to the product’s use phase as well as transportation from retail to households.

**Transportation**An additional distance of 100km for inputs and 50km for outputs was modelled except for water (no transport) and traditional cultivation processes (i.e. more accurate data was calculated). This was decided because market activities provided in Ecoinvent (accounting for most of the background processes used in these systems), include the average transportation from producer to the consumer needed to make the product available in Europe. However, an extra distance to the end consumer, in this case BAMA Fresh Cuts and the VF, was considered necessary to include the last bit of the supply chain (from the regional warehouses and retailers to the end user). Similarly, 50km were chosen for waste since the treatment of waste is assumed to be handled locally.

Distances referring to distribution were calculated using Google Maps and data from BAMA regarding market shares. It was estimated that 65% of the sold products stay south in Malmö’s region, while the remaining 35% are delivered to the Stockholm area. Thus, from Helsingborg (where the facilities of both systems are located for the baseline scenarios), 550km and 60km were used as transportation distances for mid- and north Sweden. Distribution was assumed to be outsourced to Svenska Retursystem (SRS), the most used shipping tray provider in the Swedish food industry. Hence, data regarding tray capacity and weight was obtained from the company’s website https://www.retursystem.se/sv).

The distribution-related environmental burdens attributed to the systems modelled include only the weight of the SRS trays and the bagged lettuce sent to the retailer. Other impacts, such as production and cleaning of the trays, are allocated to SRS. Transportation of consumables was modelled using the lorry process from Ecoinvent (i.e. 100% fueled by diesel) and transportation of leafy greens as lorry with refrigeration (also form Ecoinvent). No losses were attributed to this stage.

**End of Life**

Although impacts from retail were not modelled, losses in this step were considered to be 3% of the total production output for both systems. Product and packaging waste at the end of the life cycle were included since it has been shown that they can have an important role in the environmental performance of salad products (Martin et al., 2023b). Household waste was divided in two categories, namely biowaste and plastic. The first one, being 3% of the total salad at retail, was assumed to be half composted and half treated as municipal solid waste. The remaining 97% was assumed to be ingested and modelled as a cut-off. All the plastic bags were modelled as waste at the end of their life cycle, 50% being incinerated and the remaining half recycled. Motivation behind this choice was the fact that Swedish plastic recycling rates, beyond polyethylene terephthalate (PET), are relatively low and information about consumer recycling behaviour is scarce (Naturvårdsverket, 2022).

# CS: Conventional Supply

**Material inputs**

The company provided annual data regarding quantity, location, and method of cultivation for the salad products. However, due to the lack of available data for each case (e.g. field production of romaine in Germany), the crops were aggregated and modelled with existing datasets. Open-field cultivation was divided in two categories – baby spinach and the rest (i.e. iceberg, romaine, arugula) – and the datasets “spinach production - Global” and “iceberg lettuce production | iceberg lettuce | Cutoff, S” (both from Ecoinvent) were used, respectively. The polytunnel-produced crops were all modelled under the same category using the dataset from Agribalyse corresponding to “lettuce, autumn under tunnel, conventional, at farm gate/kg - France”.

The amount of water and cleaning agents employed was given and packaging material used to bring in consumables was derived from waste data granted. Cardboard was assumed to be equal to the total output (by-product) plus an extra 3% to account for potential losses (sent to residual waste) and the plastic quantity was considered to be half of that of cardboard. Wood pellets to bring raw material in was modelled assuming that only 10% of them are replaced per year and the rest are reused.

Data concerning packaging used for bagging the leafy greens was derived from information provided by the company regarding packaging sizes, (plastic) weight per bag and annual yield (of the four considered crops). It was assumed this packaging comes from 100% virgin raw materials. \\

**Infrastructure and Maintenance**

This category intends to represent BAMA’s facility in Helsingborg. Data was taken from inventory lists provided by the company and complemented with a site visit. The quantities were aggregated into higher categories to comply with the non-disclosure agreement. Different assumptions to account for lifetime were made to distribute environmental impacts of infrastructure over its entire useful life and are reflected in Table S1.

The modelled facility was assumed to be constructed for the sole purpose of serving the company’s operations and thus, the building envelope was included in this category. The interior was modelled with four lines for cutting and cleaning the raw materials and seven for bagging; machinery weighing around 6400kg and 1900kg was modelled for each four and seven lines respectively.
 To complete the inventory, some additional polyethylene pipes and pumps were added to the washing lines. Electronics (i.e. to account for items such as scales or computers), control units and robotics (e.g. electric motors) for all lines were also modelled. Additional amounts of steel, aluminium, and polyethylene were included to take a conservative approach and account for trays, bins, tanks, and tables, among others. For most cases, “market for” was considered representative enough. For the additional amount of steel and aluminium modelled however, assembly was included by adding the process “metal working, average for metal product manufacturing”. Maintenance was included in this category and quantified by expert consultation (i.e. Anna Fråne and Michael Martin).

Table S1: Lifetime assumptions for CS.

| Process | Lifetime (years) |
| --- | --- |
| Aluminium | 40 |
| Steel | 40 |
| Tubing (PE) | 5 |
| Assembly | 40 |
| Plastic | 10 |
| Control Units | 10 |
| Pumps | 10 |
| Other (electronics) | 10 |
| Cables | 10 |
| Robotics | 10 |
| Machines | 20 |

**Energy**The total amount of energy used annually (electricity and heat) was given by BAMA and modelled using Ecoinvent processes, namely Swedish market for electricity (which uses IEA data from 2018) and market for district heating.

**Transportation**The cultivation processes were modelled as “production of” instead of “market for” because the different transportation distances were considered an important aspect of the study. According to BAMA, although a significant number of raw materials are cultivated in Sweden (mostly during summer), imports are still crucial to satisfy demand and are brought mostly from Spain and Italy (but also Poland, France, and Germany). The kilometres of transportation corresponding to the different crops were calculated using Google Maps and aggregated by cultivation practice taking into account the production shares among leafy green types (for further information refer to EXCEL).

Although BAMA Fresh Cuts also supplies Finish and Baltic retailers (i.e. where SRS cannot be hired for), the share of exported products is significantly smaller than the amount of product sold nationally. Thus, it was assumed that all products were sold to the Swedish market using SRS and following the logic explained above.

**Waste Handling**

Waste generated during cultivation and transportation of raw materials (to the Helsingborg facility) was excluded. However, discarded products at the facility were modelled according to data provided by the collaborating party. The largest waste share (i.e., the water used for cleaning purposes) was modelled as wastewater sent to an external treatment plant and biowaste (28% of the plant raw material) as input for biogas production. Residual and wood waste were considered to be incinerated and the generated hazardous waste treated externally. Cardboard and plastic film (unsuitable bagging material) were not modelled as waste but as coproducts (and lastly considered negligible). The reason behind this choice being that BAMA Fresh Cuts is able to sell these products and thus they are outputs with economic value greater than zero. Packaging and plant waste from households was modelled as explained above.

# VFS: Vertical Farm Supply

**Material Inputs**

Several material inputs, or consumables, are required to run the VF. These include the growing media, seeds, water, fertilisers, CO_2_ enrichment, PH control, cleaning supplies, and packaging materials for inputs and outputs (e.g. cardboard and plastics). For the water usage including irrigation and cleaning, tap water was assumed to be employed (no rainwater collection modelled). Moreover, although a base may also be used, only nitric acid was modelled as PH adjust since water is already basic. The CO_2_ input to enhance plant growth was assumed to be purchased from Linde Industrial Gases (Martin et al., 2023b). The company has a facility in Norrköping in which they produce ethanol from grain fermentation. Since the liquid CO_2_ is considered a by-product from this process, it is modelled to account only for 50% of impacts given by the Econivent “market for carbon dioxide, liquid | carbon dioxide, liquid | Cutoff, S – RER”. Regarding packaging, consumables were assumed to be wrapped in polyethylene or cardboard (quantities taken from Ljusgårda). Data concerning bags used for sale was derived from the packaging sizes and weight information from BAMA Fresh Cuts to allow for a better comparison of both systems. The total annual yield of the VF (i.e. 520 tonnes) was assumed to be bagged and transported to retail. The values for the other material inputs including growing media, seeds and fertilisers were taken from Martin et al. (2023b) without any manipulation.

**Electricity**

The total quantity of electricity used was taken from Ljusgårda and the electricity mix composition taken as given by Ecoinvent 3.8.

**Infrastructure and Maintenance**

For the assessment of the VF, infrastructure is an important input. It can have a relevant influence in several impact categories such as climate change, freshwater eutrophication, and acidification, accounting for about 10% of the total impact in each of them (Martin et al., 2023a; Martin et al., 2023b). This category includes steel structures, tanks, trays, pumps, electronics such as control units and sensors, machinery, HVAC equipment, and LED armatures, among others. Different assumptions to account for lifetime were made to distribute environmental impacts of infrastructure over its entire useful life. Following (Martin et al., 2023c), passive elements such as steel and aluminium structures were assumed to have longer lifetimes (i.e 45 years). Life years for active equipment ranged from 2 years in the case of plastic elements to 20 in the case of machinery. LED armatures were assumed to last 5 years and pumps 10 years. The quantities for infrastructure and maintenance were again taken from Ljusgårda for the sake of consistency but aggregated into higher categories to keep the model simple.

It was assumed that the building of the VF was built in a pre-existing structure and thus, the building envelope was not included. Although renovations may have been needed, they were considered a cut-off. The motivation for this were the findings of Fnais et al. (2022), which show that the operational stage is the major determinant of the building’s resulting emissions and resource consumption. For most cases, “market for” was considered representative enough. However, for LED armatures and other growing structures, assembly of the different elements was included by adding the process “section bar extrusion, aluminium” and “metal working, average for metal product manufacturing”, respectively. Different assumptions to account for lifetime were made to distribute environmental impacts of infrastructure over its entire useful life and are reflected in Table S2.

Table S2: Lifetime assumptions for VFS.

| Process | Lifetime (years) |
| --- | --- |
| LEDs | 5 |
| Aluminium | 45 |
| Steel | 45 |
| Plastic | 2 |
| Assembly | 45 |
| Polypropylene | 10 |
| Control Units | 10 |
| Pumps | 10 |
| Other/Sensors | 10 |
| Cables | 10 |
| Machines | 20 |
| Robotics | 10 |

**Transportation**

Transportation distances for consumables and distribution of the product were modelled using the same logic as for System I. It is worth noting that the packaging process for this system is assumed to occur at the same VF; thus, no transportation is modelled to BAMA’s site. The motivation behind this choice is the fact that no cleaning is required for VF products to be labelled as “ready-to-eat” since the cultivation process does not use soil (i.e. hydroponics). Although no available literature was found regarding this fact, empirical evidence gathered was considered to suffice - Ljusgårda products are labelled as RTE in stores, and they are bagged at their VF in Tibro.

**Waste Handling**

For this product system there are two main sources of waste: the VF and the consumer. At the vertical farm the biggest amount (in kg) of waste comes from wastewater. All the tap water used for cleaning purposes is assumed to be sent to an external treatment plant together with the 2% of irrigation water not taken up by the plants. Biowaste is another significant category, and it includes discarded plants (i.e. a 3% of the total production output) and substrate used (i.e. peat and coir). It is assumed to be composted. Plastic from packaging (i.e. consumables and 1% of bags) is considered to be incinerated and plastic from infrastructure (e.g. tubing, LEDs) recycled. Electronic waste is assumed to be sent to an external treatment facility. The quantities for these categories were modelled using the total amount used to operate the VF corrected over its associated lifetime. Cardboard (from packaging) and scrap metal (from infrastructure) were assumed to be recycled. Household waste was modelled following the same logic as for System I.

# Scenario Development: Regional Electricity mixes

The electricity mixes for the Helsingborg and Luleå scenarios were modelled using the software SimaPro 9.5.0.1 and the process “Electricity, high voltage {SE}| market for electricity, high voltage | Cut-off, U” from Ecoinvent 3.8. The default composition was adapted with the shares of each generation source given by the Swedish Energy Agency and the regional mixes with the shares given by Papageorgiou et al. (2020) and Swedish Energy Agency (2023). Then, the processes regarding voltage transformation (from high to medium and from medium to low) and the market processes for (medium and low voltage) electricity were also modified using the new high voltage process modelled as initial input.

Table S3: Electricity mix composition of the four bidding areas of the Swedish electricity system in 2018. Data from Papageorgiou et al. (2020) and Swedish Energy Agency (2023).

| **Source** | **SE1** | **SE2** | **SE3** | **SE4** | **National** |
| --- | --- | --- | --- | --- | --- |
| **Nuclear** | 0% | 0% | 77% | 0% | 43% |
| **Thermal** | 1% | 2% | 6% | 20% | 5% |
| **Hydro** | 91% | 83% | 10% | 16% | 41% |
| **Solar** | 0% | 0% | 0% | 1% | 0% |
| **Wind** | 7% | 15% | 7% | 60% | 11% |
| **Unespecified** | 0% | 0% | 0% | 3% | 0% |

# Sensitivity analysis: Cultivation dataset

The sensitivity of the results to the source of the CS life cycle inventory chosen was analysed since neither primary nor secondary data was attainable regarding environmental impacts of each crop per cultivation method and this life-cycle stage contributed a significant amount to the system’s impacts. On the one hand, the open field cultivation process which represented 78% of the CS production (including iceberg, romaine and arugula) was modified from the Ecoinvent process “iceberg lettuce production | iceberg lettuce | Cutoff, S” to an Agribalyse’s alternative, namely “lettuce, open-field, conventional, at farm gate/kg – France”. For the polytunnel cultivated crops conforming 15% of total CS production, the baseline process was “lettuce, autumn under tunnel, conventional, at farm gate/kg – France” (Agribalyse) and the sensitivity was analysed using “Lettuce360 production, in heated greenhouse | lettuce | Sensitivity Cutoff, S” (Ecoinvent). The remaining 7%, being this OF production of baby spinach, was excluded.

# Results: Characterisation Tables

Below are further details of the results for the CS and VFS scenarios:

Table S4: Characterisation results for the baseline scenario of CS.

| Row Labels | Cultivation and Cleaning | Packaging | Energy | Infrastructure | Maintenance | Transport | Waste - BAMA | Waste - EoL |
| --- | --- | --- | --- | --- | --- | --- | --- | --- |
| Acidification (mol H+ eq) | 1.58E-03 | 9.92E-04 | 1.94E-04 | 8.97E-05 | 1.44E-06 | 5.17E-03 | 1.46E-04 | 5.88E-05 |
| Climate Change (kg CO_2_ eq) | 2.01E-01 | 2.24E-01 | 3.59E-02 | 1.28E-02 | 3.33E-04 | 1.45E+00 | 7.91E-02 | 9.72E-02 |
| Ecotoxicity, Freshwater (CTUe) | 1.19E+01 | 2.80E+00 | 1.46E+00 | 5.70E-01 | 7.40E-03 | 1.64E+01 | 6.84E+00 | 3.83E-01 |
| Eutrophication, Freshwater (kg P eq) | 4.41E-05 | 5.92E-05 | 1.39E-05 | 6.88E-06 | 6.54E-08 | 1.05E-04 | 2.78E-05 | 2.60E-06 |
| Land Use (Pt) | 1.89E+00 | 1.65E+00 | 8.81E-01 | 1.99E-02 | 1.73E-03 | 9.56E+00 | 1.79E-01 | 9.59E-02 |
| Resource Use, Fossil (MJ) | 2.75E+00 | 5.83E+00 | 3.84E+00 | 2.53E-01 | 4.52E-03 | 1.89E+01 | 2.12E-01 | 1.42E-01 |
| Resource Use, Minerals and Metals(kg Sb eq) | 2.39E-06 | 1.24E-06 | 4.93E-07 | 1.37E-06 | 8.91E-09 | 6.55E-06 | 1.84E-07 | 7.49E-08 |
| Water Use (m^3^ depriv.) | 1.84E+00 | 1.57E-01 | 1.11E-01 | 4.86E-03 | 4.64E-05 | 9.30E-02 | -9.38E-01 | 5.12E-03 |

Table S5: Characterisation results for the baseline scenario of VFS.

| Row Labels | Material Inputs | Fertilisers | Packaging | Energy | Infrastructure | Maintenance | Transport | Waste - Farm | Waste - EoL |
| --- | --- | --- | --- | --- | --- | --- | --- | --- | --- |
| Acidification (mol H+ eq) | 2.85E-04 | 1.25E-04 | 8.80E-04 | 2.06E-03 | 7.61E-04 | 2.64E-06 | 4.56E-04 | 6.27E-04 | 5.88E-05 |
| Climate Change (kg CO_2_ eq) | 9.27E-02 | 1.66E-02 | 2.01E-01 | 4.46E-01 | 1.42E-01 | 5.10E-04 | 1.41E-01 | 3.30E-02 | 9.72E-02 |
| Ecotoxicity, Freshwater (CTUe) | 9.82E-01 | 2.50E+00 | 2.40E+00 | 2.01E+01 | 3.53E+00 | 1.98E-02 | 1.60E+00 | 2.83E+00 | 3.83E-01 |
| Eutrophication, Freshwater (kg P eq) | 2.78E-05 | 4.04E-06 | 5.21E-05 | 1.79E-04 | 4.80E-05 | 2.44E-07 | 9.74E-06 | 4.44E-06 | 2.60E-06 |
| Land Use (Pt) | 2.95E-01 | 3.67E+00 | 6.94E-01 | 1.29E+01 | 1.61E-01 | 2.18E-03 | 1.16E+00 | 1.78E-01 | 9.59E-02 |
| Resource Use, Fossil (MJ) | 2.08E+00 | 1.98E-01 | 5.40E+00 | 5.88E+01 | 3.62E+00 | 6.64E-03 | 1.97E+00 | 2.33E-01 | 1.42E-01 |
| Resource Use, Minerals and Metals (kg Sb eq) | 1.44E-06 | 4.08E-07 | 1.04E-06 | 7.65E-06 | 6.58E-06 | 5.06E-08 | 5.74E-07 | 1.23E-07 | 7.49E-08 |
| Water Use (m^3^ depriv.) | 4.89E-01 | 8.31E-03 | 1.45E-01 | 1.69E+00 | 5.99E-02 | 1.14E-04 | 9.33E-03 | -6.14E-02 | 5.12E-03 |
